# Reconstruction and analysis of a *Kluyveromyces marxianus* genome-scale metabolic model

**DOI:** 10.1186/s12859-019-3134-5

**Published:** 2019-11-06

**Authors:** Simonas Marcišauskas, Boyang Ji, Jens Nielsen

**Affiliations:** 1 Department of Biology and Biological Engineering, Chalmers University of Technology, Kemivägen 10, SE412 96, Gothenburg, Sweden; 2 Novo Nordisk Foundation Center for Biosustainability, Technical University of Denmark, DK2800 Lyngby, Denmark; 3 BioInnovation Institute, Ole Måløes Vej 3, DK2200 Copenhagen N, Denmark

**Keywords:** Genome-scale metabolic model, *Kluyveromyces marxianus*, Thermotolerant yeast, Constraint-based flux analysis

## Abstract

**Background:**

*Kluyveromyces marxianus* is a thermotolerant yeast with multiple biotechnological potentials for industrial applications, which can metabolize a broad range of carbon sources, including less conventional sugars like lactose, xylose, arabinose and inulin. These phenotypic traits are sustained even up to 45 °C, what makes it a relevant candidate for industrial biotechnology applications, such as ethanol production. It is therefore of much interest to get more insight into the metabolism of this yeast. Recent studies suggested, that thermotolerance is achieved by reducing the number of growth-determining proteins or suppressing oxidative phosphorylation. Here we aimed to find related factors contributing to the thermotolerance of *K. marxianus*.

**Results:**

Here, we reported the first genome-scale metabolic model of *Kluyveromyces marxianus,* iSM996, using a publicly available *Kluyveromyces lactis* model as template. The model was manually curated and refined to include the missing species-specific metabolic capabilities. The iSM996 model includes 1913 reactions, associated with 996 genes and 1531 metabolites. It performed well to predict the carbon source utilization and growth rates under different growth conditions. Moreover, the model was coupled with transcriptomics data and used to perform simulations at various growth temperatures.

**Conclusions:**

*K. marxianus* iSM996 represents a well-annotated metabolic model of thermotolerant yeast, which provides a new insight into theoretical metabolic profiles at different temperatures of *K. marxianus*. This could accelerate the integrative analysis of multi-omics data, leading to model-driven strain design and improvement.

## Background

The ascomycetous yeast *Kluyveromyces marxianus* is a prospective microbial host for industrial biotechnology. It is known as a fast growing, Crabtree negative organism, capable of utilizing a broad selection of sugars and having a high secretion capacity for proteins [1, 2]. For these reasons, *K. marxianus* has been successfully applied in numerous studies for the endogenous enzymes production including inulinase [3], β-galactosidase [4], β-glucosidase [5] and β-xylosidase [6]. Even though its sister species *Kluyveromyces lactis* contains 94% of *K. marxianus* genes [7], only *K. marxianus* is thermotolerant, growing up to 45–50 °C. Since it has been isolated mainly from dairy products for years, it has GRAS (Generally Regarded as Safe) and QPS (Qualified Presumption of Safety) status, therefore making it suitable for applications in the food and pharma industry.

Thermotolerant hosts may positively impact the high temperature industrial bioprocesses in several ways. Firstly, thermotolerance is favourable once the simultaneous saccharification and fermentation (SSF) is considered as many amylases and cellulases are more efficient at high temperatures [8]. Secondly, the maintenance costs may be reduced due to the lower cost for bioreactor cooling and sterilization of cultivation media [8, 9]. The media sterility is less important at high temperatures due to the decreased competition between the species for the available medium resources. This is particularly beneficial for the complex media like whey and slurry. At high temperatures, thermotolerant yeasts are forced to reorganize their profiles at the transcriptional and proteomic levels [10]. For instance, protein folding, phosphorylation and cell wall organisation processes were upregulated in YPD medium under high temperature, thereby suppressing carbohydrate, amino acid and lipid metabolism [11, 12]. The cell likely benefits from the medium richness, while depleting biosynthetic pathways for the several YPD compounds. However, not much is known about *K. marxianus* intracellular metabolite levels at high temperatures. Such knowledge may be useful for minimal media design and for identifying high temperature metabolic capabilities, in particular for evaluation of the production potential of various compounds.

Genome-scale metabolic models (GEMs) are valuable systems biology tools as they combine genome annotation, cultivation and other experimental data for a given organism into a whole-cell metabolic network. Complementary to the general omics analysis, GEMs give an ability to integrate omics data into metabolic networks and predict the condition specific metabolic capabilities. GEMs have been successfully used to design strains and evaluate the cell capabilities upon different conditions [13, 14].

To evaluate the metabolic capabilities of *K. marxianus,* we reconstructed iSM996, the first genome-scale metabolic model (GEM) for this species. This model was used to predict the growth on various carbon sources and we found a good consistency between the model predictions and the experimental data reported in the literature. We used transcriptomics data to obtain the context specific models at different chemostat conditions, i.e. 30 °C YPD shaking, 30 °C YPD non-shaking and 45 °C YPD shaking. The models revealed the main metabolic bottlenecks associated with growth at high temperatures. To our knowledge, this approach is one of the first attempts to use GEMs to evaluate metabolic profiles at different temperatures.

## Methods

### Model reconstruction

The genome-scale metabolic network reconstruction for *K. marxianus* DMKU3–1042 was based on the published genome annotation (NCBI accession PRJDA65233) and other databases including KEGG [15], MetaCyc [16], TransportDB [17] and BRENDA [18]. Two draft models were generated using the RAVEN Toolbox [19]. The first model contained homologous reactions from iOD907, the GEM for the sister species *Kluyveromyces lactis* [20]. This was accomplished with the RAVEN function *getModelFromHomology*, which utilises BLASTP [21] for the bi-directional homology search. The following homology criteria were used: e-value 1E-30; identity 40%; alignment length of 200. An additional check to identify homologs was considered for proteins shorter than 250 amino acids, since for those it was less likely to satisfy e-value and alignment length criteria. Spontaneous iOD907 reactions without gene associations were also checked to be added to this draft model. The second draft model was generated from KEGG using the RAVEN function *getKEGGModelForOrganism*. This function uses HMMER (http://hmmer.org/) in homology search, where the query proteome is queried against KEGG Orthology (KO) specific hidden Markov model (HMM) sets. The default e-value threshold was used, i.e. 1E-50. It was then compartmentalised with the RAVEN function *predictLocalization*, which uses WoLF PSORT [22] protein scores as input. Manual curation was then performed for metabolite names and reaction reversibility, directionality, when using iOD907, Yeast 7.6 [23] and HMR2 [24] as reference. Thereafter, the unique reactions from the second draft model were then manually added to the first draft model. All the compartmentalisation discrepancies were fixed according to the latter model.

### Biomass composition

The biomass composition was adapted from iOD907 and adjusted with *K. marxianus* bibliographic data wherever possible. The cell dry weight composition (g/gDW) for protein, carbohydrate, lipid, RNA and DNA content was incorporated from cultivation study [25]. The stoichiometric coefficients for biomass compounds were then scaled-up to constitute up to 1 g of biomass in biomass equation. The experimental data from the cell wall study [26] was used to estimate the composition of the cell wall carbohydrates, namely glucan, mannan and chitin. Knowing the total carbohydrates mass in 1 g of biomass, we calculated the remaining mass for other carbohydrates (trehalose and amylose). The molar ratio between trehalose and amylose was considered the same as in iOD907. Nucleotide, deoxynucleotide and amino acid composition was calculated from genome, transcriptome (including tRNA and rRNA) and proteome according to the suggested protocol [13]. The detailed biomass composition is shown in Additional file 4. The energy parameters for the growth associated maintenance (GAM), the non-growth associated maintenance (NGAM) and P/O ratio were retained the same as in iOD907, since related information was lacking for *K. marxianus*.

### Manual curation

The semi-automatic gap filling was performed to ensure that the draft model could produce all biomass components in Verduyn medium [27]. This medium was also considered as the minimal medium in this study. The main gap-filling reaction sources were iOD907, Yeast 7.6 models and KEGG database. The semi-automatic gap filling was done for each biomass component separately, i.e. for all reactants in biomass, protein, carbohydrate, fatty acid, lipid, DNA and RNA pseudo reactions (r_1906-r_1912). The RAVEN function *fillGaps* was used to identify the candidate reactions for the gap filling, which were kept in the model upon no contradictions in the literature. Such pre-processed draft model was then greatly improved with *K. marxianus* literature knowledge. For instance, we added the missing uptake pathways for several carbon [28] sources like inulin, L-arabinose or D-mannitol. The draft model was then thoroughly curated for gene associations, EC numbers [29], metabolite names, reactions elemental/charge balance. The existing gene-protein-reaction (GPR) rules were checked for the substrate, co-factor usage and sub-cellular localization relevance. Upon any discrepancies for a particular gene, its association would be migrated to the newly created reaction with altered substrate, co-factor usage or different compartmentalization for involved metabolites. The reactions which after such curation no longer contained GPR rules, were considered for removal, unless they were found to be essential for simulations done in this study. We considered Yeast 7.6 [23], BRENDA [18] and MetaCyc [16], TransportDB [17] as reference. The additional information for every reaction is included in Additional file 2 (RXNS sheet, NOTE column).

### Model validation

The iSM996 model (Additional file 1) was checked for the overall consistency using memote, the genome-scale metabolic model test suite [30]. The full report is included as Additional file 3. The model was validated using the constraint-based flux balance analysis (FBA) [31] upon minimal medium. An ability to predict growth upon various carbon and nitrogen sources was tested and compared with the literature knowledge. During the testing of the different nitrogen sources D-glucose was chosen as a carbon source. The iSM996 was also checked for the growth rate accuracy in minimal media including various uptake rates for carbon sources. In such simulations, the lower and upper bounds for relevant substrates were set to the corresponding experimental uptake rate values. No cultivation experiments were considered in this study, see the complete reference list for the cited experimental values in Additional file 4 (Sheet 8).

### Transcriptomic data integration

The quantitative transcription start site sequencing (TSS-seq) [32] data was used to deactivate reactions (Additional file 5) and obtain condition specific models. TSS-seq data was used from Gene Expression Omnibus (GEO, https://www.ncbi.nlm.nih.gov/geo/) under the accession ID GSE66600. Three conditions were considered from the downloaded dataset: 30 °C YPD shaking (30D), 30 °C YPD non-shaking (30DS) and 45 °C YPD shaking (45D). TSS-seq dataset also contained the data for D-xylose medium (YPX) in 30 °C (30X). However, it was decided not to include this condition in the analysis, since *KmXKS1* gene, responsible for D-xylulose phosphorylation, was inactive therefore preventing D-xylose assimilation. However, all four conditions were utilised to identify the genes which were turned off in at least one condition. Thereafter, three condition specific models were obtained, each having inactivated reactions once their associated genes were inactive. No threshold values for expression abundance were considered, the gene was called as inactive if it had zero expression values in all three replicates per condition.

### Reduced cost analysis

The values for reduced cost were calculated using the *optimizeCbModel* function from The COBRA Toolbox for MATLAB [33].

## Results and discussion

### Model reconstruction

The model reconstruction of *K. marxianus* included four steps: i) obtain and curate the draft reconstruction based on template model iOD907; ii) fetch and curate the second draft model from KEGG; iii) merge both draft models; iv) perform the thorough manual curation. The first draft model after curation had 875 genes including 20 manually identified homologs and 29 non-gene-associated spontaneous reactions. Meanwhile, the compartmentalised and curated second draft model had 524 genes. These two models were merged into a new draft model with 987 genes, 1934 reactions and 1678 metabolites. We then added exchange, biomass and ATP maintenance reactions from iOD907 and performed the growth simulation in a minimal medium. As the model could predict growth, we used the draft model as a basis for further model modifications.

The manual curation started by fixing two inconsistencies inferred from the template GEM. Firstly, in iOD907 all the cytosolic metabolites could be transported to extracellular space and had exchange reactions. Since the cell ability to import or secrete all cytosolic metabolites seemed unspecific, we commenced the manual check by using Yeast 7.6 as reference. Overall, the number of transportable cytosolic metabolites was decreased from 313 to 182 in iSM996. Another iOD907 problem was that the cell could import or secrete several fatty acid-acyl carrier protein (ACP) complexes even though the cell membrane should be impermeable for such large complexes. These complexes were therefore replaced by the corresponding free fatty acids. Correspondingly, the biosynthetic pathways were added for the known products like 2-phenylethanol, phenethyl acetate or ethyl acetate.

**Fig. 1:**
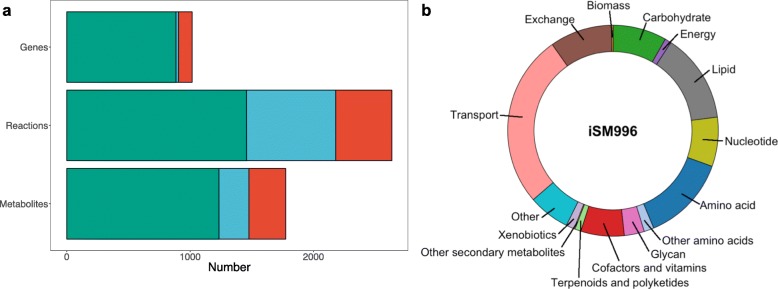
Overview of iSM996. **a** Comparison of genes, reactions and metabolites present in iSM996 and template model (iOD907). Green colour indicates overlapping entities, blue – specific to iOD907, red – specific to iSM996. **b** Distribution of reactions in each metabolic part

The iSM996 model (Fig. 1 a) features 996 genes, 1913 (including 183 exchange) reactions and 1531 metabolites thereby including *K. marxianus* specific 110 genes, 455 reactions and 298 metabolites. The most represented metabolic pathways are related to transport, exchange reactions and amino acid, lipid, carbohydrate metabolism (Fig. 1 b). Regarding transport reactions, iSM996 has 365 such reactions between the cell and extracellular space and 140 reactions between the cellular organelles. As shown in Table 1, iSM996 has the higher genome coverage than iOD907, however, it has a lower number of non-*S. cerevisiae* genes. Regarding the reactions content, iSM996 contains more reactions occurring in cytosol and less in extracellular space.

**Table 1:** Comparison between *Kluyveromyces lactis* GEM iOD907 and *Kluyveromyces marxianu*s GEM iSM996

	iOD907	iSM996
Genes	907 (17.8%)	996 (20.1%)
*S. cerevisiae* homologues	691	916
Unique	216	80
Reactions	2180	1913
Extracellular	938	507
Cytosol	853	974
Mitochondria	359	390
Endoplasmic Reticulum	30	42
Metabolites	1477	1531
Extracellular	313	191
Cytosol	822	907
Mitochondria	296	359
Endoplasmic Reticulum	46	74

### Model validation

**Fig. 2:**
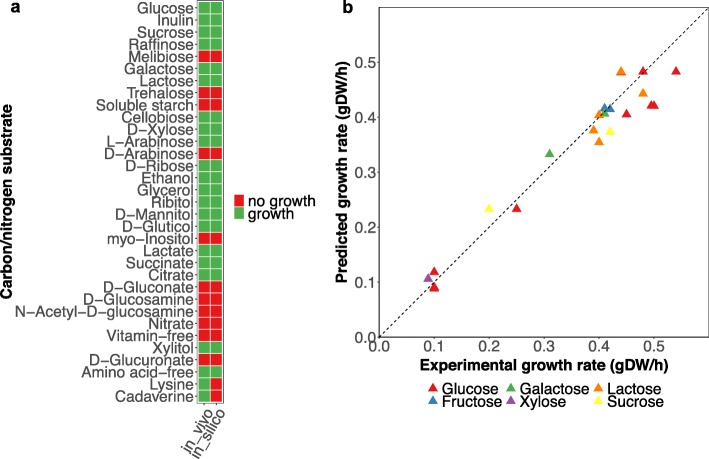
Validation results for iSM996 in minimal medium. **a** Comparison of in silico growth for various carbon and nitrogen sources with literature data. Upon simulations for nitrogen sources it was assumed that glucose is a carbon source. **b** Comparison of in silico growth rate and experimental growth rate for various carbon sources in minimal medium. The squared value of Pearson correlation coefficient between experimental and predicted growth values was 0.9445

We validated the iSM996 model by checking the growth in known carbon and nitrogen sources in minimal media (Fig. 2 a). The growth could be predicted in numerous monosaccharides (glucose, galactose, D-xylose), disaccharides (sucrose, lactose, cellobiose) and polysaccharides (inulin). The iSM996 could also predict growth in amino acid-free minimal medium, meaning that the cell is capable to de novo synthesize all the required amino acids. While in silico simulations showed growth upon all nitrogen sources, no growth could be predicted for L-lysine and cadaverine as sole carbon sources. The most likely scenario how L-lysine is catabolised and then directed to the central carbon metabolism is the 6-step linear pathway (MetaCyc LYSDEGII-PWY) occurring in *Saccharomyces cerevisiae* and providing glutarate as the final product. One remaining step would then be to convert glutarate into glutaryl-CoA, which may be further modified by known fungal enzymes. However, the LYSDEGII-PWY pathway is not associated to any *S. cerevisiae* genes, so it was impossible to run the homology search with *K. marxianus*. Meanwhile, cadaverine is the product of L-lysine decarboxylation, which does not occur in LYSDEGII-PWY. Since it is unknown how cadaverine is further catabolised in fungi, one may speculate that cadaverine is converted back to L-lysine with carbon fixation and then processed in the same hypothetical way as L-lysine. Due to a high uncertainty regarding these hypotheses, the decision was made not to include these reactions to iSM996.

An additional evaluation was performed to find the in silico explanation through the iSM996, why *K. marxianus* cannot assimilate the non-growth carbon sources, listed in Fig. 2 a. The results suggested that D-gluconate and N − acetyl−D − glucosamine cannot not be assimilated due to the absence of correspondent transporters. Melibiose, trehalose, starch, and myo-inositol lack their respective glycoside hydrolases. Unlike L-arabinose, D-arabinose cannot not be assimilated since the cell lacks the redox-driven L-arabinose isomerisation capability to D-xylulose through L-arabinitol. Finally, the growth upon D-glucosamine as a sole carbon source is not possible, since *K. marxianu*s, just like *S. cerevisiae* [34], does not have glucosamine-6-phosphate deamination capability.

The iSM996 was also checked for the growth rate prediction accuracy upon various carbon sources and their uptake rates. The results (Fig. 2 b) indicate the strong correlation between experimental and in silico growth rates. The difference between in vivo and in silico growth rates was smaller when growth rate did not exceed 0.3 h^− 1^. Whereas NGAM value has a minimal impact to predicted growth rates, one may hypothesize that in reality the cell “tweaks” the stoichiometric coefficients in the biomass equation including GAM. It is therefore not possible to re-use the same biomass composition in a wide range of the growth rates. While the biomass reactions in GEMs are usually most relevant for the growth rates lower than 0.4 h^− 1^, the more specific experimental data for the biomass composition, GAM, and NGAM are needed for the higher growth rates, if one aims to perform the simulations at high growth rates.

### In silico evaluation of *K. marxianus* metabolic capacities in stress conditions

The iSM996 model represents the whole cell metabolic capabilities assuming that all genes are active. We next used iSM996 to predict the cell metabolic stress response upon the low oxygen availability and the high temperature in the rich medium. This was done by integrating TSS-seq data to GEM and obtaining the context-specific models in YPD medium for the following conditions: 30 °C shaking (30D), 30 °C static (30DS) and 45 °C shaking (45D). The analysis would greatly benefit from additionally considering the metabolomics data, however we reckon that the comparative analysis of condition-specific models based on only transcriptomics data is still relevant to identify the key differences for biomass precursor synthesis between the conditions.

**Fig. 3:**
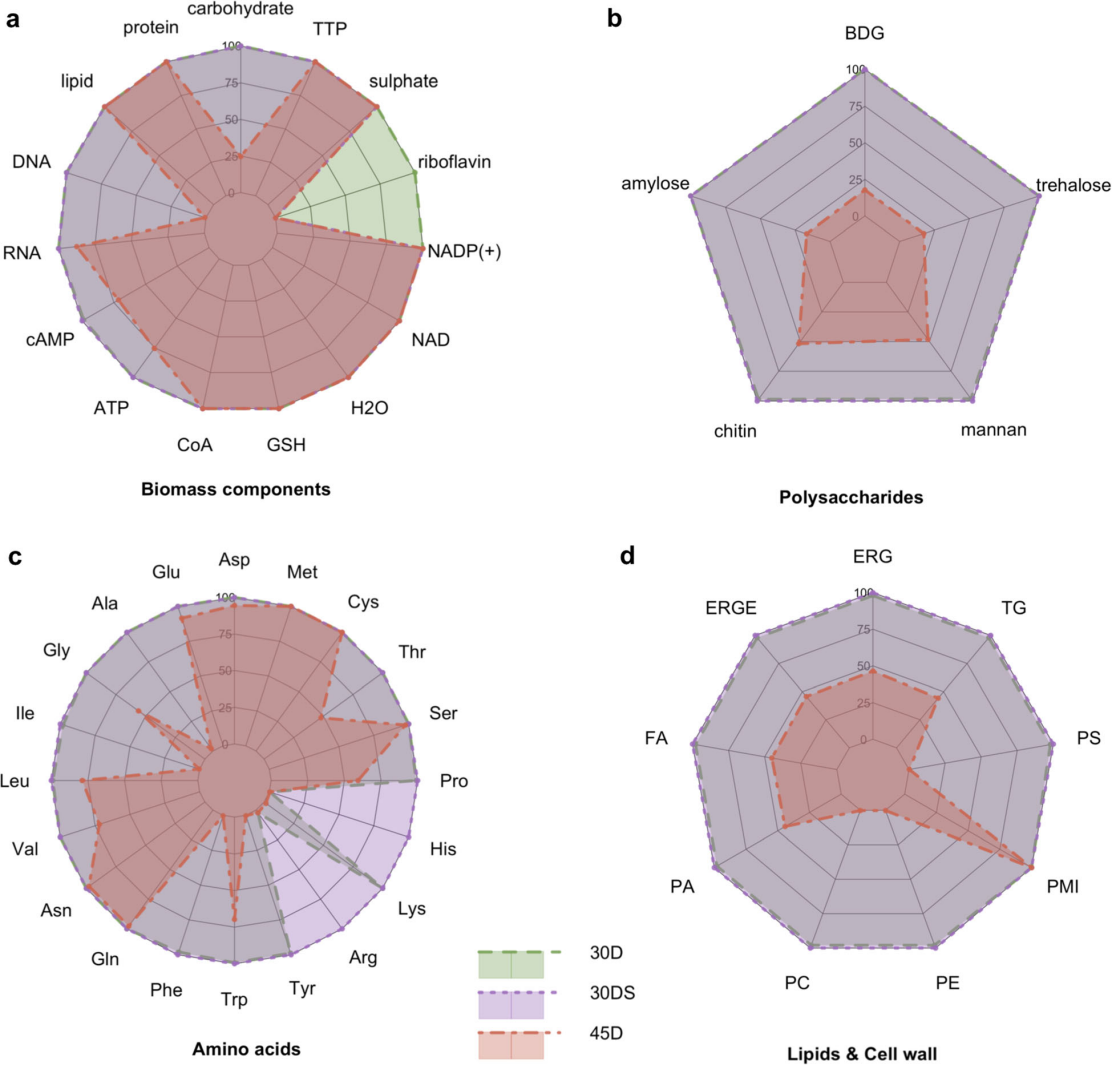
A radar chart showing the predicted potential for biomass precursors excessive production in 30D, 30DS and 45D conditions. As the magnitude is different for each metabolite, the relative production values are shown, where 100% indicates the largest production capacity between conditions. The data for the 30D condition is shown as the green polygon bordered with the dashed border while the corresponding data for the 30DS condition is in purple (dotted border) and the data for the 45D condition is in red (dot dash border) color. **a** Abbreviations: cAMP (3′,5′-cyclic AMP), CoA (coenzyme A), GSH (reduced glutathione), TTP (deoxythymidine 5′-triphosphate). **b** Abbreviations: BDG ((1- > 3)-beta-D-glucan). **c** Abbreviations (by side chain class): a) acid: Asp (L-aspartate), Glu (L-glutamate); b) aliphatic: Ala (L-alanine), Gly (glycine), Ile (L-isoleucine), Leu (L-leucine), Val (L-valine); c) amide: Asn (L-asparagine), Gln (L-glutamine); d) aromatic: Phe (L-phenylalanine), Trp (L-tryptophan), Tyr (L-tyrosine); e) basic: Arg (L-arginine), Lys (L-lysine); f) basic aromatic: His (L-histidine); g) Pro (L-proline); hydroxyl-containing: Ser (L-serine), Thr (L-threonine); h) sulphur containing: Cys (L-cysteine), Met (L-methionine). **d** Abbreviations: ergosterol (ERG), ergosterol ester (ERGE), FA (fatty acid), PA (phosphatidate), PC (phosphatidylcholine), PE (phosphatidylethanolamine), PMI (1-phosphatidyl-1D-myo-inositol), PS (phosphatidyl-L-serine), TG (triglyceride). The corresponding radar charts for precursor metabolites nucleotides are included in Additional file 6: Figure S1

The TSS-seq data was used to turn off the gene-associated reactions without transcriptional evidence while leaving the constraints for other reactions intact. These models were also used to predict the maximal production capacity for the main biomass components (Fig. 3). We calculated the excessive production above the metabolic demands for achieving 90% of the maximal growth rate. This was done by firstly fixing the lower and upper bounds for the biomass reaction to the 90% of the maximal growth rate and then maximizing the production for the biomass components of interest. TSS-seq data hinted towards a noticeably tighter metabolic regulation in 45D (80 inactivated genes) compared with 30D and 30DS (24 and 15 inactivated genes respectively). The initial FBA simulations revealed the riboflavin auxotrophy in 30DS and 45D conditions, and the ferroheme auxotrophy in the 45D condition. To enable the metabolic profile comparison between the conditions it was assumed that these metabolites are available and can be transported from the medium into the cell. Upon such consideration, the growth was predicted in all three condition-specific models. Reduced cost analysis showed that the growth was limited by L-cysteine availability and this represented a limitation in all three conditions. The high temperature condition therefore featured the most zero-flux reactions (638) while 30D and 30DS correspondingly had 544 and 541 such reactions.

The simulations indicate that across the conditions, *K. marxianus* retains the ability to allocate the amino acids necessary for roughly the same protein mass. Amino acid auxotrophies to L-arginine and L-histidine were identified in 30D and 45D. High temperature-specific auxotrophies included the previously reported L-lysine, L-isoleucine [35] and previously undiscovered auxotrophies to L-alanine, L-phenylalanine and L-tyrosine. Having the depleted biosynthetic pathways for seven amino acids at high temperatures allows the cells to conserve the precursor metabolites required for these pathways and likely contributes to an increased flux through Embden–Meyerhof–Parnas (EMP) pathway and the TCA cycle due to the increased carbon availability. However, the decreased theoretical production pool sizes in 45D for L-alanine, L-isoleucine, L-phenylalanine, L-tyrosine and L-arginine may reduce the availability for the biosynthesis of some proteins enriched by these amino acids.

**Fig. 4:**
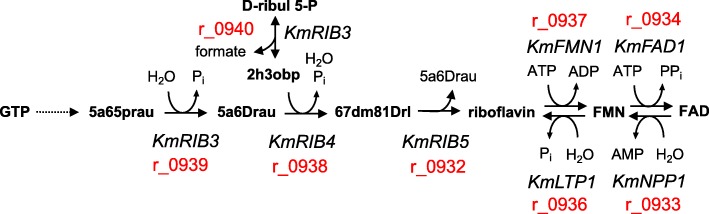
The riboflavin biosynthetic pathway. The gene names are written in italic, while the iSM996 reaction IDs are written in red colour. Abbreviations for metabolites: GTP (guanosine-5′-triphosphate), 5a65prau (5-amino-6-(5-phosphoribitylamino)uracil), 5a6Drau (5-amino-6-(D-ribitylamino) uracil, D-ribul 5-P (D-ribulose 5-phosphate), 2h3obp (2-hydroxy-3-oxobutyl phosphate), 67dm81Drl **(**6,7-dimethyl-8-(1-D-ribityl) lumazine production**)**. Abbreviations for genes: *KmRIB3* (3,4-dihydroxy-2-butanone-4-phosphate synthase), *KmRIB4* (lumazine synthase), *KmRIB5* (riboflavin synthase), *KmFMN1* (riboflavin kinase), *KmFAD1* (FAD synthetase), *KmLTP1* (putative protein phosphotyrosine phosphatase), *KmNPP1* (nucleotide pyrophosphatase/phosphodiesterase)

The auxotrophy to riboflavin, found in 30DS and 45D conditions, has the other potential benefits in addition to just conserving the building blocks. Two condition-specific strategies are considered to inactivate de novo riboflavin synthesis. As shown in Fig. 4, the main riboflavin precursors GTP (guanosine-5′-triphosphate) and D-ribul 5-P (D-ribulose 5-phosphate) are processed in their corresponding linear pathways until their final products are used to produce 67dm81Drl (6,7-dimethyl-8-(1-D-ribityl)lumazine), the immediate precursor for riboflavin. Upon the 30DS condition, the expression of *KmRIB3*, which catalyses the final reactions of GTP and D-ribul 5-P (D-ribulose 5-phosphate) linear pathways (r_0939 and r_0940 correspondingly) is inactivated. However, the interconversion ability is possibly retained between riboflavin and the downstream metabolites FMN and FAD. At the 45D condition, the expression of *KmRIB4* and *KmFMN1* genes are inactivated. The former gene is responsible for 67dm81Drl (6,7-dimethyl-8-(1-D-ribityl)lumazine) production (r_0938), while the latter gene is involved in FMN conversion from riboflavin (r_0937). Thus, under the high temperature condition, *K. marxianus* can only interconvert FMN and FAD or hydrolyse it back to riboflavin. These findings suggest that the organism has to find an optimal balance between ATP synthesis (riboflavin) and other important metabolic functions linked with FMN and FAD. While at the 30DS condition, the cell can freely allocate these three compounds. Under the 45D condition, *K. marxianus* features an irreversible riboflavin production from FMN and FAD, suggesting that ATP shortage is likely the main limitation for the cell at high temperatures, probably further affected by the limited ferroheme availability. The additional supplementation of riboflavin is known to increase the tolerance to high temperatures upon low ATP/ADP ratio [36]. Since the ATP production differences between 30 °C and 45 °C are only about 20%, one may conclude that an increased ATP demand is related to the non-growth associated maintenance processes.

The lipid pool size remains the same at all three conditions. However, this assumption is based on the arbitrary abundance value for myo-inositol (0.001 mmol/gDW/h), so one may find the different lipid pool sizes once the experimental values for myo-inositol are used. The excessive availability for the other lipid components is suppressed by an inactivation of the *KmDGK1* gene, which is a known contributor to the increased heat sensitivity when overexpressed [37].

The significant decrease of flux found in deoxynucleotides was the shortage of dAMP. Due to the inactivation of the *KmISN1* and *KmSDT1* genes, dAMP shortage cannot be compensated by salvaging other purine nucleotides. This suggest that the cell aims to conserve these building blocks. This coincides with the upregulation of the genes responsible for DNA repair [7].

## Conclusions

We hereby present iSM996, the first genome scale-metabolic model for the thermotolerant yeast *K. marxianus*. This model is capable of predicting the growth on the majority of the reported carbon and nitrogen sources. The growth simulations hereby indicate the close correspondence between in vivo an in silico growth rates for the numerous carbon sources. In addition, the model contains several unique biosynthetic pathways for aroma compounds like 2-phenylethanol, phenethyl acetate and ethyl acetate. The iSM996 was used to construct three condition specific models in YPD medium while considering the low oxygen and high temperature conditions. The results suggested that at the high temperature the cell turns off more genes thereby introducing new auxotrophies and hereby utilizing as many resources as possible from the medium. These findings may be used to the growth medium design upon the low oxygen availability and high temperature. Finally, the iSM996 model is a solid tool to evaluate *K. marxianus* metabolic features, allowing the experimental data integration and the model-driven strain design.

## Supplementary information

**Additional file 1.** Model file in SBML format.

**Additional file 2.** Model file in Excel format.

**Additional file 3.** The full iSM996 report from memote – the genome-scale metabolic model test suite.

**Additional file 4.** Biomass composition details for overall components (Sheet 1), deoxyribonucleotides (Sheet 2), ribonucleotides (Sheet 3), amino acids (Sheet 4). Also included the model performance upon chemostat conditions for the growth rate (Sheet 5), oxygen consumption (Sheet 6) and carbon dioxide production (Sheet 7). The model performance upon different carbon sources in respect of the growth rate is also included (Sheet 8). References are included in the file.

**Additional file 5.** List of inactive genes (Sheet 1) and corresponding reactions (Sheet 2) between 30D, 30DS, 30X and 45D conditions.

**Additional file 6: Figure S1.** A radar chart showing the predicted potential for biomass precursors excessive production in 30D, 30DS and 45D conditions. As the magnitude is different for each metabolite, the relative production values are shown, where 100% indicates the largest production capacity between conditions. The data for the 30D condition is shown as the green polygon bordered with the dashed border while the corresponding data for the 30DS condition is in purple (dotted border) and the data for the 45D condition is in red (dot dash border) color. (a) Abbreviations: G6P (alpha-D-glucose 6-phosphate), F6P (beta-D-fructose 6-phosphate), E4P (D-erythrose 4-phosphate), R5P (D-ribose 5-phosphate), GAP (D-glyceraldehyde 3-phosphate), 3PG (3-phosphoglycerate), PEP (phosphoenolpyruvate), PYR (pyruvate), OXA (oxaloacetate), ACA (acetyl-CoA), 2OG (2-oxoglutarate), SCA (succinyl-CoA). (b) Abbreviations: AMP (adenosine monophosphate), CMP (cytidine monophosphate), GMP (guanosine monophosphate), UMP (uridine monophosphate), dAMP (deoxyadenosine monophosphate), dCMP (deoxycytidine monophosphate), dGMP (deoxyguanosine monophosphate), dTMP (thymidine monophosphate).

## Data Availability

The iSM996 GEM together with the supporting information will be made publicly available on GitHub upon the acceptance of the paper in https://github.com/SysBioChalmers/Kluyveromyces_marxianus-GEM.

## Abbreviations

FBA: Flux balance analysis
GEM: Genome-scale metabolic model
YPD: Yeast extract peptone glucose
YPX: Yeast extract peptone xylose
